# Admission Serum Iron as an Independent Risk Factor for Postoperative Delayed Cerebral Ischemia Following Aneurysmal Subarachnoid Hemorrhage: A Propensity-Matched Analysis

**DOI:** 10.3390/brainsci12091183

**Published:** 2022-09-02

**Authors:** Yi-Bin Zhang, Feng Zheng, Lampis Stavrinou, Hao-Jie Wang, Wen-Jian Fan, Pei-Sen Yao, Yuan-Xiang Lin, Roland Goldbrunner, Shu-Fa Zheng, Pantelis Stavrinou, De-Zhi Kang

**Affiliations:** 1 Department of Neurosurgery, Neurosurgery Research Institute, The First Affiliated Hospital, Fujian Medical University, Fuzhou 350005, China; eabin.z@fjmu.edu.cn (Y.-B.Z.); haojie2016@fjmu.edu.cn (H.-J.W.); 711920@fjmu.edu.cn (W.-J.F.); peisen_yao@fjmu.edu.cn (P.-S.Y.); lyx99070@163.com (Y.-X.L.); zsf2002110@163.com (S.-F.Z.); 2 Department of Neurosurgery, National Regional Medical Center, Binhai Campus of the First Affiliated Hospital, Fujian Medical University, Fuzhou 350212, China; 3 Department of Neurosurgery, The Second Affiliated Hospital, Fujian Medical University, Quanzhou 362000, China; dr.feng.zheng@gmail.com; 4 2nd Department of Neurosurgery, “Attikon” University Hospital, Athens Medical School, National and Kapodistrian University, 999028 Athens, Greece; lampis.stavrinou@gmail.com; 5 Department of Neurosurgery, Center for Neurosurgery, Faculty of Medicine and University Hospital, University of Cologne, 50923 Cologne, Germany; roland.goldbrunner@uk-koeln.de (R.G.); panstavrinou@gmail.com (P.S.); 6 Department of Neurosurgery, Metropolitan Hospital, 999028 Athens, Greece; 7 Fujian Provincial Clinical Research Center for Neurological Disease, The First Affiliated Hospital, Fujian Medical University, Fuzhou 350005, China; 8 Fujian Provincial Institutes of Brain Disorders and Brain Sciences, The First Affiliated Hospital, Fujian Medical University, Fuzhou 350005, China; 9 Clinical Research and Translation Center, The First Affiliated Hospital, Fujian Medical University, Fuzhou 350005, China

**Keywords:** stroke, intracranial aneurysm, subarachnoid hemorrhage, delayed cerebral ischemia, hemoglobin, iron

## Abstract

This study aimed to investigate the association between serum iron (SI) and postoperative delayed cerebral ischemia (DCI) following aneurysmal subarachnoid hemorrhage (aSAH). We retrospectively analyzed 985 consecutive adult patients diagnosed with aSAH. Demographic, clinical, and laboratory data were recorded. Univariate and multivariate analyses were employed to assess the association between SI and DCI. Propensity-score matching (PSM) analysis was implemented to reduce confounding. Postoperative DCI developed in 14.38% of patients. Lower SI upon admission was detected in aSAH patients with severe clinical conditions and severe aSAH. SI was negatively correlated with WFNS grade (r = −0.3744, *p* < 0.001) and modified Fisher (mFisher) grade (r = −0.2520, *p* < 0.001). Multivariable analysis revealed lower SI was independently associated with DCI [odds ratios (OR) 0.281, 95% confidence interval (CI) 0.177–0.448, *p* < 0.001], while WFNS grade and mFisher grade were not. The receiver-operating characteristics (ROC) curve analysis of SI for DCI gave an area under the curve (AUC) of 0.7 and an optimal cut-off of 7.5 μmol/L (95% CI 0.665 to 0.733, *p* < 0.0001). PSM demonstrated the DCI group had a significantly lower SI than the non-DCI group (10.91 ± 6.86 vs. 20.34 ± 8.01 μmol/L, *p* < 0.001). Lower SI remained a significant independent predictor for DCI and an independent poor prognostic factor of aSAH in multivariate analysis (OR 0.363, 95% CI 0.209–0.630, *p* < 0.001). The predictive performance of SI for poor outcome had a corresponding AUC of 0.718 after PSM. Lower SI upon admission is significantly associated with WFNS grade, mFisher grade, and predicts postoperative DCI and poor outcome at 90 days following aSAH.

## 1. Introduction

Aneurysmal subarachnoid hemorrhage (aSAH), characterized by the extravasation of blood into the subarachnoid space caused by intracranial aneurysm rupture [1,2], is a life-threatening hemorrhagic stroke with high mortality and disability rate [3]. One major complication is the delayed cerebral ischemia (DCI), developing in approximately 22–33% of patients [4,5,6,7], between 5 and 14 days (peak at 5–7 days) after SAH [3,8], and is consistently related to adverse clinical outcome and permanent disability [9,10]. Clinical identification of DCI is often challenging since hypotension, rebleeding, infection, hypoxia, hydrocephalus, and sedatives can produce a similar clinical manifestation [8,11]. A 2013 meta-analysis identified potential predictors for DCI following aSAH, including smoking, sex, the initial loss of consciousness, hyperglycemia, history of diabetes, hydrocephalus, history of hypertension, low hemoglobin (Hb), and early systemic inflammatory response syndrome [7]. Although several hypotheses have been proposed to explain the development of DCI, the presence of red blood cells, Hb, and Hb breakdown-products in close proximity to major cerebral vessels and cerebral cortex have been closely involved with the onset, development, and pathogenesis of DCI following aSAH [12,13,14].

Iron is one of the hemoglobin degradation products, leading to arterial structural damage, microcirculatory derangement, and neurotoxicity through a series of biochemical reactions in experimental subarachnoid hemorrhage (SAH) [14,15,16]. Iron overload-mediated neurotoxicity may contribute to acute and secondary brain injury that involves delayed brain atrophy, edema, DCI, and neuronal cell damage following aSAH [15,17]. After aSAH, increased iron levels in the cerebrospinal fluid (CSF) or the brain tissue were correlated with DCI [15]. However, the need for invasive sourcing of CSF-iron limits its clinical utility as a biomarker. To date, no clinical studies have evaluated serum iron (SI) and iron-mediated neurotoxicity following aSAH.

In this study, we explored whether lower SI levels upon admission correlate with Hunt–Hess grade, modified Fisher grade (mFisher grade), and postoperative DCI.

## 2. Materials and Methods

### 2.1. Study Population

**Figure 1:**
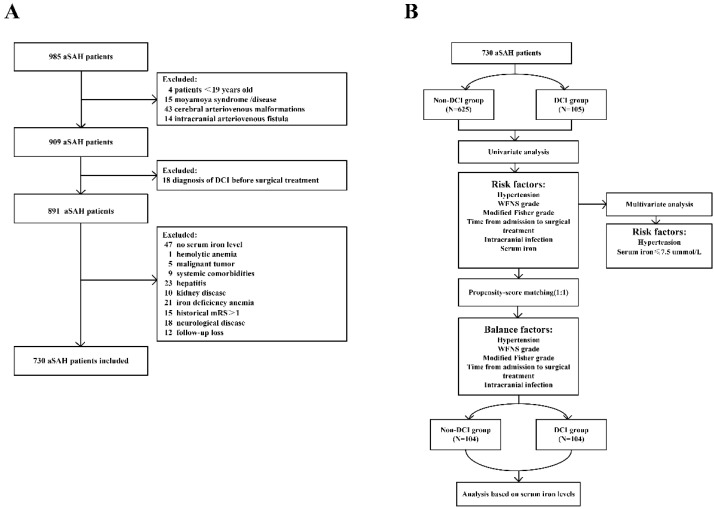
(**A**) Flow diagram of the patient selection process; (**B**) Flowchart of propensity-score matching. aSAH: aneurysmal subarachnoid hemorrhage; DCI: delayed cerebral ischemia; mRS: modified Rankin Scale; WFNS: world federation of neurosurgical societies.

We retrospectively analyzed 985 consecutive adult patients diagnosed with aSAH who were admitted in the First Affiliated Hospital of Fujian Medical University between June 2014 and June 2018. The diagnosis of SAH was established by computed tomography (CT) upon admission or by the presence of blood and xanthochromia in the cerebrospinal fluid (CSF) as per standard clinical practice. The responsible intracranial pathology was detected using computed tomography angiography (CTA) and/or digital subtraction angiography (DSA). The exclusion criteria were: (1) age younger than 19 years; (2) patients with moya-moya disease, cerebral arteriovenous malformations, or intracranial arteriovenous fistula; (3) patients diagnosed with DCI before surgical or endovascular treatment; (4) history of neurological disease or mRS > 1; (5) iron deficiency anemia; (6) no serum iron level test at admission; (7) acute kidney damage or chronic kidney disease; and (8) concurrent systemic comorbidities, including liver cirrhosis and malignancy. The detailed flow of the selection process is illustrated in Figure 1.

### 2.2. Data Collection

Patient demographic details and clinical information, including age, sex, relevant medical history, mFisher grade, World Federation of Neurosurgical Societies (WFNS) grade, aneurysm characteristics (location and size of the ruptured aneurysm), admission laboratory data, time from admission to treatment, treatment methods (clipping vs. coiling), and post-treatment complications, were recorded. Blood samples were retrieved from a peripheral vein within 1 h of arrival at our neurosurgical department before any treatment commenced. Laboratory tests included serum biochemical indexes and routine blood tests. Admission CT scans were scored using the mFisher grade and patients were divided into mild aSAH (mFisher grade 1–2) or severe aSAH (mFisher grade 3–4) [18]. Clinical neurological status at admission was evaluated using the WFNS grade and patients were categorized into severe clinical condition (WFNS grade IV–V) or mild clinical condition (WFNS grade I–III) [18].

### 2.3. Clinical Management

The ruptured aneurysms were secured either by surgical clipping or endovascular coiling as soon as possible. Nimodipine orally (60 mg Q6H) was administered to all patients. Blood pressure was continuously monitored during nimodipine administration, maintaining systolic pressure at 110–130 mmHg or the lower limit of patients’ blood pressure. Patients were given fluid therapy, and acid-base balance was monitored to avoid hypovolemia and ensure adequate cerebral perfusion pressure. Patients with DCI were treated with hemodynamic therapy (hypertension, hypervolemia) to maintain systolic blood pressure between 180 and 220 mmHg [19,20].

### 2.4. Outcome Assessment

Outcome assessments included the occurrence of DCI and functional outcomes. The diagnosis of DCI was based on a reduced level of consciousness and changes in neurological status (defined as the gradual onset of new focal neurological symptom and/or a decrease by two points on the Glasgow Coma Scale (GCS), new confirmed cerebral infarction on CT or MRI scan that was not visible on the initial or immediate post-treatment CT scan, and not attributable to other potential causes of clinical deterioration (such as rebleeding, seizures, hydrocephalus, or electrolyte imbalances) [20,21]. For the definition of DCI, the presence of transcranial doppler/angiographic vasospasm was not required [22]. The diagnosis of DCI was set by a medical doctor with experience in neuro-intensive care. In case of uncertainties, the diagnosis was discussed in a neurovascular board.

Functional outcome was assessed with the modified Rankin Scale (mRS, 0–6; 0–2 indicates a favorable outcome, whereas 3–6 indicates an unfavorable outcome) during outpatient visits or telephone interview or WeChat interview at 90 days after aSAH onset [23].

### 2.5. Statistical Analysis

Continuous variables were presented as the mean ± standard deviation (SD) or median interquartile range (IQR) values and were compared using the Student’ *t*-test and Mann–Whitney U test, as appropriate. Categorical variables were expressed as count (percentage) using the chi-square and Fisher’s exact tests. Pearson correlation analysis was performed for the positive or negative association between SI and clinical risk factors. Univariate analytical predictive findings with *p* < 0.1 were further subjected to multivariable analyses. SI was dichotomized as “≤optimal cutoff value” and “>optimal cutoff value” in the multivariate model. Moreover, the receiver-operating characteristics (ROC) curve was plotted, and the area under the curve (AUC) was calculated to assess the sensitivity and specificity of potential parameters for predicting DCI and outcome. Propensity score matching (PSM) analysis was implemented with a 1:1 nearest-neighbor matching algorithm to obtain matched pairs. In the univariate analysis, the covariates found to be significantly associated with postoperative DCI and outcome were included in the matching. Statistical analyses were performed using SPSS software version 25.0 (IBM SPSS, IBM Corp., New York, NY, USA), GraphPad Prism 8 (GraphPad Software, San Diego, CA, USA), and MedCalc version 20.0.4 (MedCalc Software, Ostend, Belgium). For all analyses, statistical significance was assigned as *p* < 0.05 (ns: *p* > 0.05, *: *p* < 0.05, **: *p* ≤ 0.01, ***: *p* ≤ 0.001, ****: *p*≤ 0.0001).

## 3. Results

### 3.1. General Characteristics

Out of a total of 985 patients with SAH, 730 patients fulfilled the inclusion criteria and were included in this study. A total of 60.28% were female and 16.6% were older than 65 years. Of these, 105 patients (14.38%) developed DCI. The primary clinical characteristics of the patients are shown in Table 1 and Figure 1. An amount of 122 (16.7%) patients were in severe clinical condition, and 184 (25.2%) patients had severe aSAH on admission. The average SI level was 12.52 ± 6.64 μmol/L. A total of 471 (64.5%) patients were treated using neurosurgical clipping, while the rest were treated endovascularly. The time from aneurysm treatment to diagnosis of DCI was 5 (3–6) days. Unfavorable functional outcome at 90 days after aSAH onset was observed in 133 (18.20%) patients.

### 3.2. Association of SI with Initial Clinical Status at Admission

**Figure 2:**
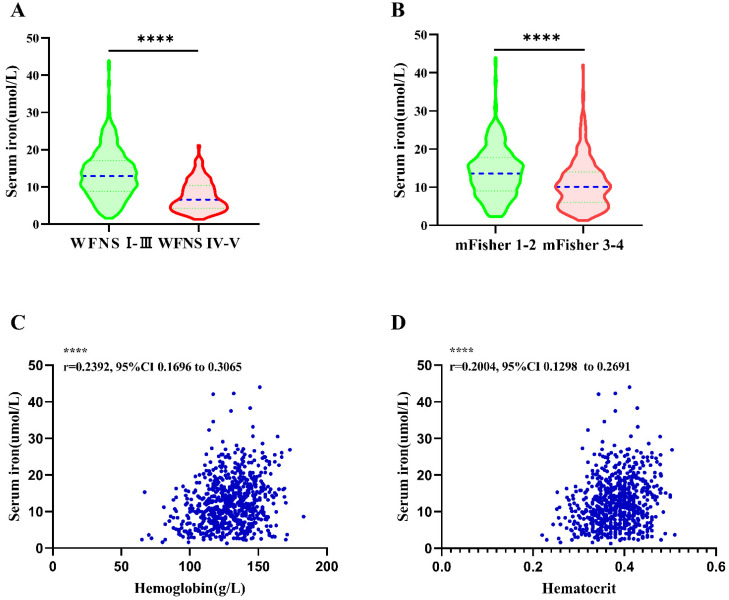
Associations between serum iron levels and initial clinical status. (**A**) Comparison of serum iron in aSAH patients between mild (WFNS grade I-III) clinical conditions and severe (WFNS grade IV–V) clinical conditions. (**B**) Serum iron levels in patients with mild (mFisher1–2) and severe (mFisher3–4) aSAH. (**C**) The correlation of serum iron levels with hemoglobin. (**D**) The correlation of serum iron levels with Hematocrit. Data were shown for violin plots in panel A and panel B. Groups were compared using Student’ *t*-test. Correlations were determined using Pearson’s correlation analysis. (****: *p* ≤ 0.0001) aSAH: aneurysmal subarachnoid hemorrhage; DCI: delayed cerebral ischemia; WFNS: world federation of neurosurgical societies; mFisher: modified Fisher.

A lower SI level was detected in patients with severe clinical condition on admission (WFNS grade IV–V) compared to those with WFNS grade I–III (7.56 ± 4.04 vs. 13.52 ± 6.62 μmol/L, *p* < 0.001 (Figure 2A). Additionally, SI level was significantly lower in aSAH patients with mFisher grade 3–4 than mFisher grade 1–2 (10.00 ± 5.20 vs. 13.37 ± 6.86 μmol/L, *p*< 0.001) (Figure 2B). Pearson correlation analysis demonstrated a significant negative correlation between SI and WFNS grade (WFNS grade r = −0.3744, R^2^ 0.1402, 95% CI = −0.4353 to −0.3103, *p* < 0.001), as well as a negative correlation between SI and mFisher grade (mFisher grade r = −0.2520, R^2^ 0.06349, 95% CI = −0.3187 to −0.1828, *p* < 0.001). Correlation analysis showed a significant positive relationship between SI and Hb and between SI and hematocrit (HCT) (Hb r = 0.2392, R^2^ = 0.05723, 95% CI = 0.1696 to 0.3065, *p* < 0.001; HCT r = 0.2004, R^2^ = 0.04018, 95% CI = 0.1298 to 0.2691, *p* < 0.001(Figure 2C,D).

### 3.3. Association of Decreased SI with Postoperative DCI

**Figure 3:**
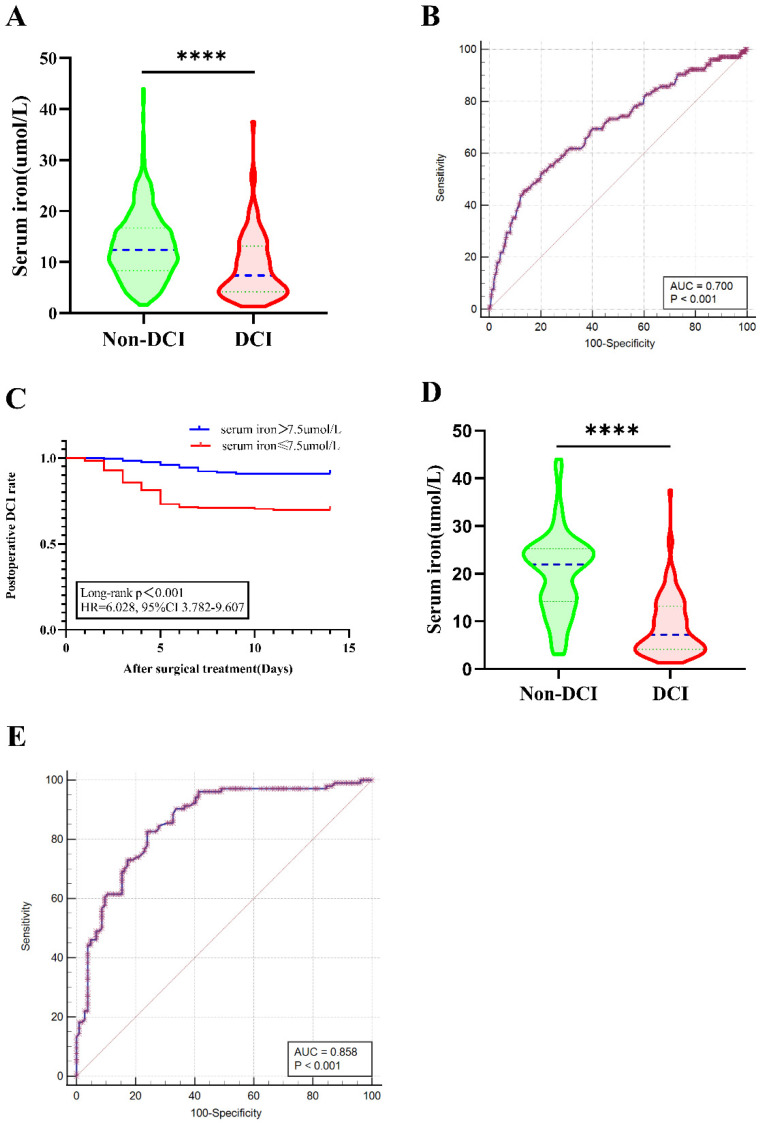
Associations between serum iron levels and DCI. (**A**) Comparison of serum iron levels in aSAH patients between DCI and non–DCI. (**B**) ROC curve analysis of serum iron as a predictor in aSAH patients for DCI gave an AUC value of 0.7 and an optimal cut-off value of 7.5 μmol/L with a corresponding of sensitivity at 52.38% and specificity of 79.68%. (**C**) Kaplan–Meier curve of the 14-day postoperative DCI–rate of aSAH by serum iron level. Log-rank test revealed that patients with serum iron ≤ 7.5 μmol/L had a significantly worse postoperative DCI than patients with >7.5 μmol/L (HR = 6.028, 95% CI 3.782–9.607, *p* < 0.001). (**D**) Comparison of serum iron levels in aSAH patients between DCI and non-DCI after PSM. (**E**) After PSM, the AUC of serum iron level was 0.858 (95% CI 0.803–0.902; *p* < 0.0001) (Sensitivity = 82.69%; Specificity = 75.96%) for postoperative DCI based on a cut–off value of 14.1 μmol/L. Data were shown for violin plots in panel A and panel D. Groups were compared using Student’ *t*-test. (****: *p* ≤ 0.0001) AUC: area under the curve; aSAH: aneurysmal subarachnoid hemorrhage; DCI: delayed cerebral ischemia; mFisher: modified Fisher; mRS: modified Rankin Scale; PSM: propensity-score matching; ROC: Receiver operating curve. WFNS: world federation of neurosurgical societies.

Seven hundred and thirty aSAH patients were categorized into either the DCI group (*n* = 105) or the non-DCI group (*n* = 625). In univariate analysis, the two groups showed significantly statistical differences in hypertension, WFNS grade, mFisher grade, time from admission to treatment, intracranial infection, and SI levels (Table 2). Patients with DCI had significantly lower SI levels than those without DCI (8.99 ± 6.26 μmol/L vs. 13.11 ± 6.52 μmol/L; *p* < 0.001) (Table 2 and Figure 3A). Admission SI levels predicted DCI development (OR = 0.232, 95% CI = 0.151–0.356; *p* < 0.001) (Table 2). To further define the predictive values of SI, multivariable logistic regression model analysis was performed using adjusted confounders including hypertension, higher WFNS grade (IV–V), poor mFisher grade (3–4), time from admission to treatment, intracranial infection, and SI. Lower SI level was independently associated with postoperative DCI (OR 0.281, 95% CI 0.177–0.448, *p* < 0.001) (Table 2). ROC curve analysis of SI as a predictor for DCI in aSAH patients gave an AUC value of 0.7 and an optimal cut-off value of 7.5 μmol/L with a corresponding sensitivity of 52.38% and specificity of 79.68% (95% CI 0.665 to 0.733, *p* < 0.0001) (Figure 3B). Cumulative postoperative DCI rate using Kaplan–Meier analysis within 14 days is presented in Figure 3C.

After PSM, there was one patient in DCI group who could not be matched. No statistical significance was detected in hypertension, WFNS grade, mFisher grade, time from admission to surgical treatment, and intracranial infection between the two groups. The matched DCI group had a significantly lower SI level than the matched non–DCI group (9.00 ± 6.29 vs. 20.13 ± 8.45 μmol/L, *p* < 0.001) (Table 1 and Figure 2D). The AUC of SI level was 0.858 (95% CI 0.803–0.902, *p* < 0.001; Sensitivity = 82.69%; Specificity = 75.96%) (Figure 3E) for postoperative DCI based on a cut-off value of 14.1 μmol/L.

### 3.4. Association of Decreased Serum Iron with 90-Day Outcome

**Figure 4:**
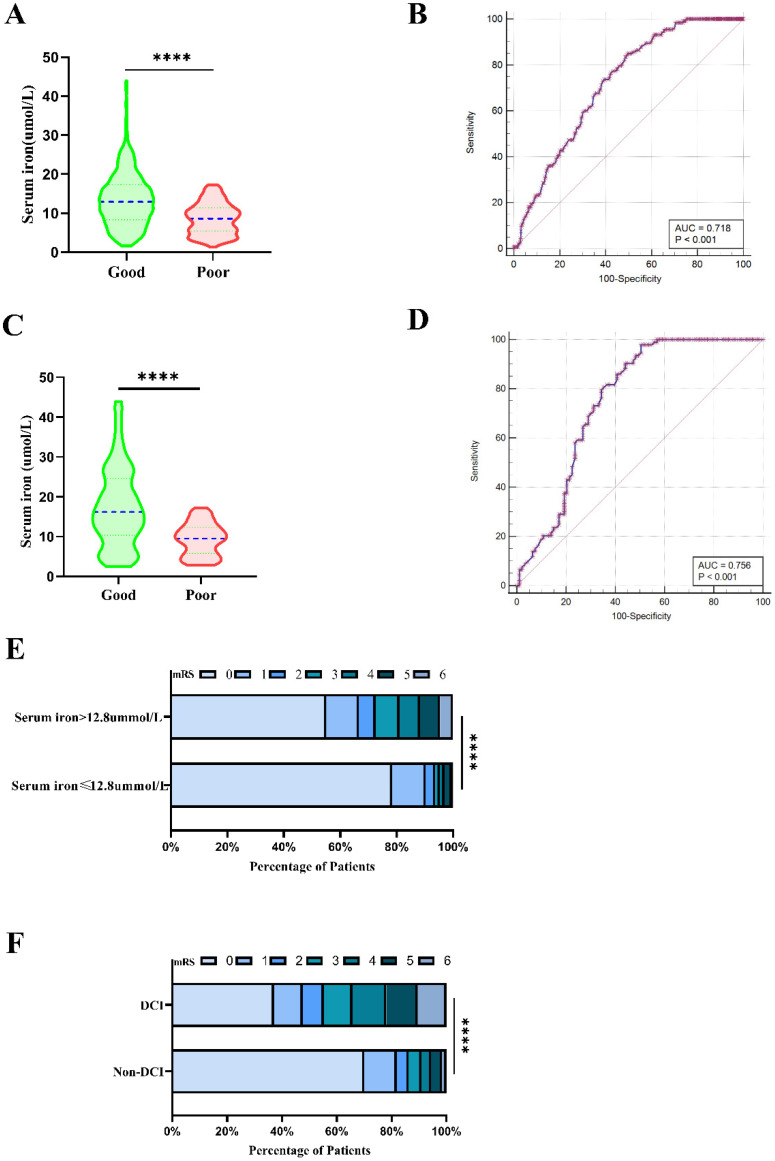
Associations of serum iron, DCI with outcome. (**A**) Comparison of serum iron levels in aSAH patients between good outcome and poor outcome. (**B**) ROC curve analysis of serum iron for predicting poor prognosis. The AUC of serum iron level was 0.718 (Sensitivity = 84.96%; Specificity = 50.25%) for poor prognosis based on the best threshold of 12.8 μmol/L. (**C**) Comparison of serum iron levels in aSAH patients between good outcome and poor outcome after PSM. (**D**) ROC curve analysis of serum iron for predicting poor prognosis after PSM. The AUC of serum iron level was 0.756 (Sensitivity = 97.85%; Specificity = 49.46%) for poor prognosis based on a cut-off value of 16.3 μmol/L. (**E**) Distribution of mRS scores at 90 days for patients with lower serum iron ≤ 12.8 μmol/L and higher serum iron > 12.8 μmol/L). The percentage of patients within each score category on the 7-point scale (0 indicates no symptoms and 6 indicates death) at 90 days. (**F**) Distribution of mRS scores at 90 days for patients with DCI and without DCI. The percentage of patients within each score category on the 7-point scale (0 indicates no symptoms and 6 indicates death) at 90 days. (****: *p* ≤ 0.0001) AUC: area under the curve; aSAH: aneurysmal subarachnoid hemorrhage; DCI: delayed cerebral ischemia; mFisher: modified Fisher; mRS: modified Rankin Scale; PSM: propensity-score matching; ROC: Receiver operating curve.

Significant statistical differences were observed in age, hypertension, WFNS grade, mFisher grade, DCI, and SI in univariate analysis (Table 3). The patients in the poor outcome group (mRS 3–6) had significantly lower SI levels than those in the good outcome group (mRS 0–2), (8.59 ± 3.96 vs. 13.40 ± 6.80 μmol/L; *p* < 0.001) (Table 3 and Figure 4A). Admission lower SI levels predicted poor outcomes for patients with aSAH (OR 0.175, 95% CI 0.106–0.289, *p* < 0.001) (Table 4). After adjusting for age, hypertension, WFNS grade, mFisher grade, DCI, admission WFNS grade, and mFisher grade, admission SI ≤ 12.8 μmol/L and postoperative DCI were associated with poor prognosis (SI OR 0.363, 95% CI 0.209–0.630, *p* < 0.001; DCI OR 0.254, 95% CI 0.149–0.434, *p* < 0.001) (Table 4). The predictive performance of SI for the outcome (the AUC was 0.718, 95% CI 0.684 to 0.751, *p* < 0.0001; the sensitivity was 84.96%, and the specificity was 50.25% based on the best threshold of 12.8 μmol/L) is illustrated by the ROC analysis (Figure 4B).

To further determine the predictive values of SI for prognosis, PSM was also employed to balance the risk factors in univariate analysis. After PSM, there was no statistical significance in age, hypertension, WFNS grade, mFisher grade, and postoperative DCI between the two groups. A lower SI level, however, still appeared as an independent risk factor of poor outcome of aSAH. The matched poor outcome correlated with significantly lower serum iron than the matched good outcome (9.38 ± 4.00 μmol/L vs.17.32 ± 9.90 μmol/L; *p* < 0.001) (Table 3 and Figure 4C). After PSM, the AUC of SI level was 0.756 (Sensitivity = 97.85%; Specificity = 49.46%) for poor prognosis based on a cut-off value of 16.3 μmol/L (Figure 4D).

Patients with a SI ≤ 12.8 μmol/L had a statistically worse prognosis than patients with a SI > 12.8 μmol/L. Figure 4E details the distribution of mRS scores for the two groups of patients. Patients with DCI had a statistically worse prognosis than patients without DCI (Table 1). mRS scores in patients with DCI and without DCI are detailed in Figure 4F.

## 4. Discussion

The present study identified the association of SI with DCI in aSAH patients. Our study demonstrated that lower SI level (≤7.5 μmol/L) was independently associated with DCI in aSAH patients. Reported risk factors for DCI after aSAH, including mFisher-grade, WFNS grade, and hypertension [6,24,25], were balanced in the present study. After PSM, a lower SI level was still considered an independent risk factor for development of DCI after aSAH; the matched DCI group had a significantly lower SI than the matched non-DCI group. Furthermore, lower SI level was significantly associated with poor admission WFNS grade and severe clinical condition at admission. Consistent with the previous findings, in the present study too, lower SI after aSAH was a predictor of unfavorable outcome [26]. Notably, the present study is the first to document the potential predictive power of decreased admission SI level as an independent risk factor for postoperative DCI following aSAH. 

Predicting the occurrence of DCI is of crucial value for aSAH management. Previous reports have demonstrated that total hemorrhage volume is a risk factor for DCI in aSAH patients [27,28]. The fresh blood leaks from the wall of the ruptured aneurysm into the subarachnoid space, distributes irregularly over the entire brain, and penetrates deeper layers of the cortex following aSAH. As erythrocytes lyse, the brain is exposed to elevated concentration of hemoglobin (Hb) [29]. Iron, a primary degradation product of Hb, is also degraded on the brain cortex. SAH causing iron overload in the brain has been observed three weeks after aneurysmatic bleeding and remained elevated during the 1-year follow-up neuroimaging [15]. In experimental animal models, manipulation of brain tissue by inserting a microdialysis catheter was found to cause microhemorrhage along with biochemical changes, which may explain the higher iron levels in the initial stage of cerebral hemorrhage [30]. Evidence suggested that elevated iron levels in the CSF were associated with DCI and the development of deep cerebral infarcts following SAH [31]. 

Several previous studies [15,31,32] indicated that increased iron levels were correlated with DCI in aSAH patients [31]. Our study found that lower SI level (≤7.5 μmol/L) was independently associated with postoperative DCI following aSAH. In contrast to prior studies [31,32,33], we measured the iron levels from serum samples rather than directly from brain tissue or CSF. In a relevant study, Anil Can et al. reported [34] that lower SI was significantly associated with intracranial aneurysm rupture. We found that aSAH patients with severe clinical conditions or poor mFisher grade at admission had a low SI level. Iron accumulation in the brain led to oxidative stress and neuronal cell damage, including arterial structural damage, microcirculatory derangement, and neurotoxicity, through radical formation secondary to Fenton reactions following SAH [14,15,35,36]. Our results imply that different underlying mechanisms might exist between SI and the development of postoperative DCI.

A meta-analysis of fifty-two studies on 33 potential predictors for DCI following aSAH indicated hyperglycemia, hydrocephalus, history of hypertension, and systemic inflammatory response syndrome as potential predictors [7]. Systemic inflammatory response syndrome is common [37] and is considered a fundamental factor that increases susceptibility to DCI after aSAH [38,39]. Numerous inflammatory biomarkers, including C-reactive protein (CRP) [40], neutrophil to lymphocyte ratio (NLR) [25,41], white blood cell count (WBC) [40], interleukin-6 (IL-6) [42], and tumor necrosis factor-α (TNF-α), were presented at elevated levels in plasma following aSAH, and were related to the development of DCI [38,39,43]. 

Prior work documented a consistent link between inflammatory cytokines and iron regulation and metabolism in neurological diseases, indicating that disturbance of iron metabolism driven by cytokine modification at the acute phase contributed to a reduction of SI [44]. Experimental data in internal cerebral hemorrhage (ICH) illustrated that free iron is involved in inflammatory reaction after ICH [29,45]. In general, SI concentration is usually decreased in inflammatory diseases of humans and animals [46,47], and decreased serum iron levels in critically ill patients correlate with the inflammatory status and length of stay in intensive care patients [48]. Lower SI was associated with increased CRP in patients with severe renal disease [49]. The present study demonstrated that a reduced SI level is detected in aSAH patients with DCI, thus supporting a possible link between iron metabolism, inflammation, and postoperative DCI.

Although our study found a correlation between SI levels and DCI, the exact pathophysiological background of this correlation is unclear. Besides the already mentioned link between iron metabolism and inflammation, other mechanisms may also play a role. Previous studies have found a correlation between reduced iron levels and hyperglycemia, which in turn has been associated with DCI after aSAH [7,50].

In the present study, we also found a significant positive relationship between SI and Hb. Iron deficiency reduces the amount of hemoglobin, thereby decreasing the amount of oxygen in the blood, leading to insufficient oxygen delivery to the brain. This can cause hypoxia in the brain, ultimately leading to ischemic stroke [51]. Ayling et al. reported that lower hemoglobin is associated with DCI in patients with aSAH [52]. Furthermore, lower SI levels enhance red blood cell oxidative stress [53]. Oxidative stress is associated with multiple secondary complications, such as micro thrombosis, neuronal apoptosis, and the release of reactive oxygen species [54]. Numerous clinical and experimental studies have demonstrated that iron-mediated oxidative stress aggravates cerebral vasospasm and DCI following aSAH [31,55,56,57].

Several limitations in this study need to be acknowledged. Firstly, the study is limited by its retrospective nature, particularly with regard to determining DCI. Due to the complex, multifactorial, and unpredictable clinical process in aSAH patients, it is still difficult to accurately set the diagnosis of DCI. Furthermore, we used a single-point method to measure laboratory data such as SI at admission; however, the SI concentration may change with time. Further study is needed to determine whether changes in serum levels over time are related to the onset of DCI. Finally, several inflammation-based biomarkers, such as IL-6, CRP, NLR, and WBC, have been associated with postoperative DCI in aSAH patients [25,40]. Unfortunately, given that inflammatory biomarkers were not incorporated in the present study, we are unable to provide such a comparison.

## 5. Conclusions

Lower SI level at admission is significantly associated with WFNS grade, mFisher grade, and may predict postoperative DCI and poor functional outcome at 90 days for aSAH patients.

## Figures and Tables

**Table 1:** Univariate analysis of association with DCI before and after propensity-score matching.

	Before Propensity-Score Matching			After Propensity-Score Matching		
Characteristics	Non-DCI	DCI	*p* Value	Non-DCI	DCI	*p* Value
	(*n* = 625)	(*n* = 105)		(*n* = 104)	(*n* = 104)	
Age, yrs, mean ± SD	54.72 ± 11.47	55.65 ± 11.42	0.440	55.34 ± 11.22	55.76 ± 11.42	0.788
Gender (*n*, %)			0.513			1.00
Male	235 (37.6)	43 (41.0)		43 (41.3)	43 (41.3)	
Female	390 (62.4)	62 (59.0)		61 (58.7)	61 (58.7)	
Smoking (*n*, %)	112 (17.9)	16 (15.2)	0.504	23 (22.1)	16 (15.4)	0.214
Alcohol (*n*, %)	62 (9.9)	8 (7.6)	0.459	9 (8.7)	8 (7.7)	0.800
Medical history						
Hypertension (*n*, %)	307 (49.1)	75 (71.4)	<0.001	81 (77.9)	74 (71.2)	0.218
Diabetes (*n*, %)	68 (10.9)	13 (12.4)	0.650	15 (14.4)	13 (12.4)	0.685
Coronary heart disease (*n*, %)	19 (3.0)	2 (1.9)	0.520	6 (5.8)	2 (1.9)	0.149
Hyperlipidemia (*n*, %)	119 (19.0)	15 (14.3)	0.244	25 (24.0)	15 (14.4)	0.079
WFNS grade			<0.001			0.114
I–III	535 (85.6)	73 (69.5)		82 (78.8)	72 (69.2)	
IV–V	90 (14.4)	32 (30.5)		22 (21.2)	32 (30.8)	
Modified Fisher grade			<0.001			0.078
1–2	482 (77.1)	64 (61.0)		75 (72.1)	63 (60.6)	
3–4	143 (22.9)	41 (39.0)		29 (27.9)	41 (39.4)	
Aneurysm characteristics (*n*, %)						
Multiple aneurysms	115 (18.4)	23 (21.9)	0.396	17 (16.3)	23 (22.1)	0.291
Single aneurysm location			0.206			0.146
Anterior cerebral artery	33 (6.5)	2 (2.4)		7 (18.0)	2 (2.5)	
Anterior communicating artery	164 (32.2)	30 (36.6)		32 (36.8)	29 (35.8)	
Internal carotid artery	76 (14.9)	8 (9.8)		15 (17.2)	8 (9.9)	
Middle cerebral artery	104 (20.4)	24 (29.3)		14 (16.1)	24 (29.6)	
Posterior communicating artery	109 (21.4)	16 (19.5)		15 (17.2)	16 (19.8)	
Others	24 (4.7)	2 (2.4)		4 (4.6)	2 (2.5)	
Aneurysm size (*n*, %)			0.405			0.487
<5 mm	265 (42.4)	41 (39.0)		47 (45.2)	41 (39.4)	
5–15 mm	299 (47.8)	53 (50.5)		48 (46.2)	52 (50.0)	
15–25 mm	51 (8.2)	7 (6.7)		8 (7.7)	7 (6.7)	
>25 mm	10 (1.6)	4 (3.8)		1 (1.0)	4 (3.8)	
Surgical methods (*n*, %)			0.132			0.059
Clipping	399 (63.8)	75 (71.4)		61 (58.7)	74 (71.2)	
Coiling	226 (36.2)	30 (28.6)		43 (41.3)	30 (28.8)	
Time from admission to surgical treatment (*n*, %)			0.018			0.874
≤72 h	396 (63.4)	79 (75.2)		77 (74.0)	78 (75.0)	
>72 h	229 (36.6)	26 (24.8)		27 (26.0)	26 (25.0)	
Intracranial infection (*n*, %)	45 (7.2)	15 (14.3)	0.014	18 (17.3)	14 (13.5)	0.442
Hemoglobin, g/L, mean ± SD	128.73 ± 17.97	130.13 ± 18.63	0.475	134.32 ± 17.14	129.88 ± 18.53	0.074
Hematocrit, %, mean ± SD	38.19 ± 4.86	38.56 ± 4.88	0.473	39.47 ± 4.74	38.49 ± 4.85	0.144
Serum iron, μmol/L, mean ± SD	13.11 ± 6.52	8.99 ± 6.26	<0.001	20.13 ± 8.45	9.00 ± 6.29	<0.001
modified Rankin Scale (*n*, %)			<0.001			<0.001
0–2	539 (86.2)	58 (55.2)		94 (90.4)	57 (54.8)	
3–6	86 (13.8)	47 (44.8)		10 (9.6)	47 (45.2)	

aSAH: aneurysmal subarachnoid hemorrhage; DCI: delayed cerebral ischemia mFisher: modified Fisher; SD: standard deviation; WFNS: World Federation of Neurosurgical Societies.

**Table 2:** Predictors for DCI of aSAH in multivariate model.

	Unadjusted OR (95% CI)				Adjusted AOR (95% CI)			
Independent Variable	OR	Lower	Upper	*p* Value	OR	Lower	Upper	*p* Value
Hypertension	0.386	0.246	0.607	<0.001	0.452	0.281	0.726	0.001
WFNS grade (IV-V)	2.606	1.626	4.176	<0.001	0.791	0.456	1.369	0.402
Modified Fisher grade (3–4)	0.463	0.300	0.715	0.001	0.736	0.449	1.206	0.223
Time from admission to surgical treatment(≤72 h)	0.569	0.355	0.912	0.019	1.289	0.781	2.126	0.320
Intracranial infection	2.148	1.150	4.014	0.017	1.832	0.940	3.571	0.075
Serum iron (≤7.5 μmol/L)	0.232	0.151	0.356	<0.001	0.281	0.177	0.448	<0.001

aSAH: aneurysmal subarachnoid hemorrhage; DCI: delayed cerebral ischemia mFisher: modified Fisher; SD: standard deviation; WFNS: World Federation of Neurosurgical Societies.

**Table 3:** Univariate analysis of association with outcome in aSAH before and after propensity-score matching.

	Before Propensity-Score Matching			After Propensity-Score Matching		
Characteristics	Good Outcome	Poor Outcome	*p* Value	Good Outcome	Poor Outcome	*p* Value
	(*n* = 597)	(*n* = 133)		(*n* = 93)	(*n* = 93)	
Age, yrs, mean ± SD	54.17 ± 11.30	57.89 ± 11.69	0.001	56.53 ± 11.54	56.75 ± 11.57	0.894
Gender (*n*, %)			0.898			1.00
Male	228 (38.2)	50 (37.6)		34 (36.6)	34 (36.6)	
Female	369 (61.8)	83 (62.4)		59 (63.4)	59 (63.4)	
Smoking (*n*, %)	110 (18.4)	18 (10.5)	0.180	20 (21.5)	13 (14.0)	0.179
Alcohol (*n*, %)	60 (10.1)	10 (7.5)	0.356	10 (10.8)	7 (7.5)	0.445
Medical history						
Hypertension (*n*, %)	293 (49.1)	89 (66.9)	<0.001	66 (71.0)	61 (65.6)	0.431
Diabetes (*n*, %)	63 (10.6)	18 (13.5)	0.322	13 (14.0)	11 (11.8)	0.662
Coronary heart disease (*n*, %)	16 (2.7)	5 (3.8)	0.501	1 (1.1)	2 (2.2)	0.561
Hyperlipidemia (*n*, %)	105 (17.6)	29 (21.8)	0.256	20 (21.5)	23 (34.7)	0.602
WFNS grade			<0.001			1.00
I–III	543 (90.1)	65 (48.9)		60 (64.5)	60 (64.5)	
IV–V	54 (9.1)	68 (51.1)		33 (35.5)	33 (35.5)	
Modified Fisher grade			<0.001			0.305
1–2	487 (81.6)	59 (44.4)		44 (47.3)	51 (54.8)	
3–4	110 (18.4)	74 (55.6)		49 (52.7)	42 (45.2)	
Aneurysm characteristics (*n*, %)						
Multiple aneurysms	108 (18.1)	30 (22.6)	0.234	16 (17.2)	22 (23.7)	0.275
Single aneurysm location			0.196			0.648
Anterior cerebral artery	28 (5.3)	7 (6.8)		5 (6.5)	4 (5.6)	
Anterior communicating artery	158 (32.3)	36 (35.0)		27 (35.1)	25 (35.2)	
Internal carotid artery	63 (12.9)	21 (20.4)		8 (10.4)	13 (18.3)	
Middle cerebral artery	107 (21.9)	21 (20.4)		16 (20.8)	16 (22.5)	
Posterior communicating artery	109 (22.3)	16 (15.5)		18 (23.4)	12 (16.9)	
Others	24 (4.9)	2 (1.9)		3 (3.9)	1 (1.4)	
Aneurysm size (*n*, %)			0.570			0.594
<5 mm	246 (41.2)	60 (45.1)		37 (39.8)	44 (47.3)	
5–15 mm	292 (48.9)	60 (45.1)		50 (53.8)	41 (44.1)	
15–25 mm	49 (8.2)	9 (6.8)		4 (4.3)	6 (6.5)	
>25 mm	10 (1.7)	4 (3.0)		2 (2.2)	4 (2.2)	
Surgical methods (*n*, %)			0.260			0.117
Clipping	396 (63.3)	78 (58.6)		68 (73.1)	58 (62.4)	
Coiling	201 (33.7)	55 (41.4)		25 (26.9)	35 (37.6)	
Time from admission to surgical treatment (*n*, %)			0.370			0.633
≤72 h	384 (64.3)	91 (68.4)		66 (71.0)	63 (67.7)	
>72 h	213 (35.7)	42 (31.6)		27 (29.0)	30 (32.3)	
Intracranial infection (*n*, %)	48 (8.0)	12 (9.0)	0.709	8 (8.6)	7 (7.5)	0.788
Hemoglobin, g/L, mean ± SD	128.47 ± 17.69	131.02 ± 19.57	0.168	130.63 ± 16.48	130.71 ± 19.83	0.978
Hematocrit, %, mean ± SD	38.12 ± 4.81	38.79 ± 5.05	0.165	38.44 ± 4.43	38.74 ± 5.00	0.664
Serum iron, μmol/L, mean ± SD	13.40 ± 6.80	8.59 ± 3.96	<0.001	17.32 ± 9.90	9.38 ± 4.00	<0.001
DCI (*n*, %)	58 (9.7)	47 (35.3)	<0.001	23 (24.7)	22 (23.7)	0.864

aSAH: aneurysmal subarachnoid hemorrhage; DCI: delayed cerebral ischemia mFisher: modified Fisher; SD: standard deviation; WFNS: World Federation of Neurosurgical Societies.

**Table 4:** Predictors for outcome of aSAH in multivariate model.

	Unadjusted				Adjusted			
		OR (95% CI)				AOR (95% CI)		
Independent Variable	OR	Lower	Upper	*p* Value	OR	Lower	Upper	*p* Value
Age	1.029	1.012	1.047	0.001	1.024	1.004	1.045	0.020
Hypertension	0.476	0.321	0.707	<0.001	0.868	0.536	1.406	0.564
WFNS grade (IV–Ⅴ)	0.095	0.061	0.148	<0.001	0.178	0.107	0.295	<0.001
mFisher grade (3–4)	0.180	0.121	0.269	<0.001	0.394	0.245	0.635	<0.001
DCI	0.197	0.126	0.308	<0.001	0.254	0.149	0.434	<0.001
Serum iron (≤12.8 μmol/L)	0.175	0.106	0.289	<0.001	0.363	0.209	0.630	<0.001

aSAH: aneurysmal subarachnoid hemorrhage; DCI: delayed cerebral ischemia mFisher: modified Fisher; SD: standard deviation; WFNS: World Federation of Neurosurgical Societies.

## Data Availability

The raw data supporting the conclusions of this article will be made available by the authors without undue reservation.
