# Correction: Zhang et al. Admission Serum Iron as an Independent Risk Factor for Postoperative Delayed Cerebral Ischemia Following Aneurysmal Subarachnoid Hemorrhage: A Propensity-Matched Analysis. *Brain Sci.* 2022, *12*, 1183

**DOI:** 10.3390/brainsci12111438

**Published:** 2022-10-26

**Authors:** Yi-Bin Zhang, Feng Zheng, Lampis Stavrinou, Hao-Jie Wang, Wen-Jian Fan, Pei-Sen Yao, Yuan-Xiang Lin, Roland Goldbrunner, Shu-Fa Zheng, Pantelis Stavrinou, De-Zhi Kang

**Affiliations:** 1 Department of Neurosurgery, Neurosurgery Research Institute, The First Affiliated Hospital, Fujian Medical University, Fuzhou 350005, China; 2 Department of Neurosurgery, National Regional Medical Center, Binhai Campus of the First Affiliated Hospital, Fujian Medical University, Fuzhou 350212, China; 3 Department of Neurosurgery, The Second Affiliated Hospital, Fujian Medical University, Quanzhou 362000, China; 4 2nd Department of Neurosurgery, “Attikon” University Hospital, Athens Medical School, National and Kapodistrian University, 999028 Athens, Greece; 5 Department of Neurosurgery, Center for Neurosurgery, Faculty of Medicine and University Hospital, University of Cologne, 50923 Cologne, Germany; 6 Department of Neurosurgery, Metropolitan Hospital, 999028 Athens, Greece; 7 Fujian Provincial Clinical Research Center for Neurological Disease, The First Affiliated Hospital, Fujian Medical University, Fuzhou 350005, China; 8 Fujian Provincial Institutes of Brain Disorders and Brain Sciences, The First Affiliated Hospital, Fujian Medical University, Fuzhou 350005, China; 9 Clinical Research and Translation Center, The First Affiliated Hospital, Fujian Medical University, Fuzhou 350005, China

We would like to submit the following corrections to our recently published paper [1].

## Error in the Text

The sentence correction on Page 8 needs correction:

In the original sentence, it was:

The matched DCI group had a significantly lower SI level than the matched non–DCI group (10.91 ± 6.86 vs. 20.34 ± 8.01 μmol/L, *p* < 0.001) (Table 1 and Figure 2D).

The correct sentence should be: 

The matched DCI group had a significantly lower SI level than the matched non–DCI group (9.00 ± 6.29 vs. 20.13 ± 8.45 μmol/L, *p* < 0.001) (Table 1 and Figure 2D).

## Errors in Tables

In the original publication, there was one mistake in Table 1 and two mistakes in Table 3. In Table 1, the number of patients from admission to surgical treatment (≤72 h) in the Non-DCI group was 77 after PSM. In Table 3, the number of patients with WFNS grade (IV–V) was 68 in the poor outcome before PSM, and the number of patients from admission to surgical treatment (≤72 h) in the good outcome group was 66 after PSM. The corrected Table 1 and Table 3 appear below.

**Table 1:** Univariate analysis of association with DCI before and after propensity-score matching.

	Before Propensity-Score Matching			After Propensity-Score Matching		
Characteristics	Non-DCI	DCI	*p* Value	Non-DCI	DCI	*p* Value
	(*n* = 625)	(*n* = 105)		(*n* = 104)	(*n* = 104)	
Age, yrs, mean ± SD	54.72 ± 11.47	55.65 ± 11.42	0.440	55.34 ± 11.22	55.76 ± 11.42	0.788
Gender (*n*, %)			0.513			1.00
Male	235 (37.6)	43 (41.0)		43 (41.3)	43 (41.3)	
Female	390 (62.4)	62 (59.0)		61 (58.7)	61 (58.7)	
Smoking (*n*, %)	112 (17.9)	16 (15.2)	0.504	23 (22.1)	16 (15.4)	0.214
Alcohol (*n*, %)	62 (9.9)	8 (7.6)	0.459	9 (8.7)	8 (7.7)	0.800
Medical history						
Hypertension (*n*, %)	307 (49.1)	75 (71.4)	<0.001	81 (77.9)	74 (71.2)	0.218
Diabetes (*n*, %)	68 (10.9)	13 (12.4)	0.650	15 (14.4)	13 (12.4)	0.685
Coronary heart disease (*n*, %)	19 (3.0)	2 (1.9)	0.520	6 (5.8)	2 (1.9)	0.149
Hyperlipidemia (*n*, %)	119 (19.0)	15 (14.3)	0.244	25 (24.0)	15 (14.4)	0.079
WFNS grade			<0.001			0.114
I–III	535 (85.6)	73 (69.5)		82 (78.8)	72 (69.2)	
IV–V	90 (14.4)	32 (30.5)		22 (21.2)	32 (30.8)	
Modified Fisher grade			<0.001			0.078
1–2	482 (77.1)	64 (61.0)		75 (72.1)	63 (60.6)	
3–4	143 (22.9)	41 (39.0)		29 (27.9)	41 (39.4)	
Aneurysm characteristics (*n*, %)						
Multiple aneurysms	115 (18.4)	23 (21.9)	0.396	17 (16.3)	23 (22.1)	0.291
Single aneurysm location			0.206			0.146
Anterior cerebral artery	33 (6.5)	2 (2.4)		7 (18.0)	2 (2.5)	
Anterior communicating ar tery	164 (32.2)	30 (36.6)		32 (36.8)	29 (35.8)	
Internal carotid artery	76 (14.9)	8 (9.8)		15 (17.2)	8 (9.9)	
Middle cerebral artery	104 (20.4)	24 (29.3)		14 (16.1)	24 (29.6)	
Posterior communicating artery	109 (21.4)	16 (19.5)		15 (17.2)	16 (19.8)	
Others	24 (4.7)	2 (2.4)		4 (4.6)	2 (2.5)	
Aneurysm size (*n*, %)			0.405			0.487
<5 mm	265 (42.4)	41 (39.0)		47 (45.2)	41 (39.4)	
5–15 mm	299 (47.8)	53 (50.5)		48 (46.2)	52 (50.0)	
15–25 mm	51 (8.2)	7 (6.7)		8 (7.7)	7 (6.7)	
>25 mm	10 (1.6)	4 (3.8)		1 (1.0)	4 (3.8)	
Surgical methods (*n*, %)			0.132			0.059
Clipping	399 (63.8)	75 (71.4)		61 (58.7)	74 (71.2)	
Coiling	226 (36.2)	30 (28.6)		43 (41.3)	30 (28.8)	
Time from admission to surgical treatment (*n*, %)			0.018			0.874
≤72 h	396 (63.4)	79 (75.2)		77 (74.0)	78 (75.0)	
>72 h	229 (36.6)	26 (24.8)		27 (26.0)	26 (25.0)	
Intracranial infection (*n*, %)	45 (7.2)	15 (14.3)	0.014	18 (17.3)	14 (13.5)	0.442
Hemoglobin, g/L, mean ± SD	128.73 ± 17.97	130.13 ± 18.63	0.475	134.32 ± 17.14	129.88 ± 18.53	0.074
Hematocrit, %, mean ± SD	38.19 ± 4.86	38.56 ± 4.88	0.473	39.47 ± 4.74	38.49 ± 4.85	0.144
Serum iron, umol/L, mean ± SD	13.11 ± 6.52	8.99 ± 6.26	<0.001	20.13 ± 8.45	9.00 ± 6.29	<0.001
modified Rankin Scale (*n*, %)			<0.001			<0.001
0–2	539 (86.2)	58 (55.2)		94 (90.4)	57 (54.8)	
3–6	86 (13.8)	47 (44.8)		10 (9.6)	47 (45.2)	

aSAH: aneurysmal subarachnoid hemorrhage; DCI: delayed cerebral ischemia mFisher: modified Fisher; SD: standard deviation; WFNS: World Federation of Neurosurgical Societies.

**Table 3:** Univariate analysis of association with outcome in aSAH before and after propensity-score matching.

	Before Propensity-Score Matching			After Propensity-Score Matching		
Characteristics	Good Outcome	Poor Outcome	*p* Value	Good Outcome	Poor Outcome	*p* Value
	(*n* = 597)	(*n* = 133)		(*n* = 93)	(*n* = 93)	
Age, yrs, mean ± SD	54.17 ± 11.30	57.89 ± 11.69	0.001	56.53 ± 11.54	56.75 ± 11.57	0.894
Gender (*n*, %)			0.898			1.00
Male	228 (38.2)	50 (37.6)		34 (36.6)	34 (36.6)	
Female	369 (61.8)	83 (62.4)		59 (63.4)	59 (63.4)	
Smoking (*n*, %)	110 (18.4)	18 (10.5)	0.180	20 (21.5)	13 (14.0)	0.179
Alcohol (*n*, %)	60 (10.1)	10 (7.5)	0.356	10 (10.8)	7 (7.5)	0.445
Medical history						
Hypertension (*n*, %)	293 (49.1)	89 (66.9)	<0.001	66 (71.0)	61 (65.6)	0.431
Diabetes (*n*, %)	63 (10.6)	18 (13.5)	0.322	13 (14.0)	11 (11.8)	0.662
Coronary heart disease (*n*, %)	16 (2.7)	5 (3.8)	0.501	1 (1.1)	2 (2.2)	0.561
Hyperlipidemia (*n*, %)	105 (17.6)	29 (21.8)	0.256	20 (21.5)	23 (34.7)	0.602
WFNS grade			<0.001			1.00
I–III	543 (90.1)	65 (48.9)		60 (64.5)	60 (64.5)	
IV–V	54 (9.1)	68 (51.1)		33 (35.5)	33 (35.5)	
Modified Fisher grade			<0.001			0.305
1–2	487 (81.6)	59 (44.4)		44 (47.3)	51 (54.8)	
3–4	110 (18.4)	74 (55.6)		49 (52.7)	42 (45.2)	
Aneurysm characteristics (*n*, %)						
Multiple aneurysms	108 (18.1)	30 (22.6)	0.234	16 (17.2)	22 (23.7)	0.275
Single aneurysm location			0.196			0.648
Anterior cerebral artery	28 (5.3)	7 (6.8)		5 (6.5)	4 (5.6)	
Anterior communicating artery	158 (32.3)	36 (35.0)		27 (35.1)	25 (35.2)	
Internal carotid artery	63 (12.9)	21 (20.4)		8 (10.4)	13 (18.3)	
Middle cerebral artery	107 (21.9)	21 (20.4)		16 (20.8)	16 (22.5)	
Posterior communicating artery	109 (22.3)	16 (15.5)		18 (23.4)	12 (16.9)	
Others	24 (4.9)	2 (1.9)		3 (3.9)	1 (1.4)	
Aneurysm size (*n*, %)			0.570			0.594
<5 mm	246 (41.2)	60 (45.1)		37 (39.8)	44 (47.3)	
5–15 mm	292 (48.9)	60 (45.1)		50 (53.8)	41 (44.1)	
15–25 mm	49 (8.2)	9 (6.8)		4 (4.3)	6 (6.5)	
>25 mm	10 (1.7)	4 (3.0)		2 (2.2)	4 (2.2)	
Surgical methods (*n*, %)			0.260			0.117
Clipping	396 (63.3)	78 (58.6)		68 (73.1)	58 (62.4)	
Coiling	201 (33.7)	55 (41.4)		25 (26.9)	35 (37.6)	
Time from admission to surgical treatment (*n*, %)			0.370			0.633
≤72 h	384 (64.3)	91 (68.4)		66 (71.0)	63 (67.7)	
>72 h	213 (35.7)	42 (31.6)		27 (29.0)	30 (32.3)	
Intracranial infection (*n*, %)	48 (8.0)	12 (9.0)	0.709	8 (8.6)	7 (7.5)	0.788
Hemoglobin, g/L, mean ± SD	128.47 ± 17.69	131.02 ± 19.57	0.168	130.63 ± 16.48	130.71 ± 19.83	0.978
Hematocrit, %, mean ± SD	38.12 ± 4.81	38.79 ± 5.05	0.165	38.44 ± 4.43	38.74 ± 5.00	0.664
Serum iron, umol/L, mean ± SD	13.40 ± 6.80	8.59 ± 3.96	<0.001	17.32 ± 9.90	9.38 ± 4.00	<0.001
DCI (*n*, %)	58 (9.7)	47 (35.3)	<0.001	23 (24.7)	22 (23.7)	0.864

aSAH: aneurysmal subarachnoid hemorrhage; DCI: delayed cerebral ischemia mFisher: modified Fisher; SD: standard deviation; WFNS: World Federation of Neurosurgical Societies.

The authors apologize for any inconvenience caused and state that the scientific conclusions are unaffected. This correction was approved by the Academic Editor. The original publication has also been updated.
